# Innovative cross-layer defense mechanisms for blackhole and wormhole attacks in wireless ad-hoc networks

**DOI:** 10.1038/s41598-025-97094-0

**Published:** 2025-04-28

**Authors:** Jagadeesan Srinivasan

**Affiliations:** Department of Software and Systems Engineering, School of Computer Science Engineering and Information Systems, Vellore Institute of Technology, Vellore, Tamil Nadu India

**Keywords:** Cross-layer, Enhanced-SVM, Blackhole attack, Wormhole attack, Wireless ad-hoc networks, Computer science, Engineering, Mathematics and computing

## Abstract

Wireless ad-hoc networks operate independently of existing infrastructure, using devices like access points to connect end-user computing devices. Current methods face issues such as low detection accuracy, structural deviation, and extended processing times. This paper proposes a cross-layer approach that leverages knowledge from the physical and Medium Access Control (MAC) layers, which is then shared with higher layers to effectively mitigate wormhole and blackhole attacks. A wormhole attack disrupts communication through tunneling, while a blackhole attack manipulates network traffic by impersonating the source. The proposed cross-layer framework integrates the network, MAC, and physical layers, and is independent of specific network protocols. The physical layer handles channel interference, the network layer manages process handling, and the MAC layer oversees bandwidth information and tracks failed transmissions. Performance metrics are measured in seconds. The Enhanced Support Vector Machine (E-SVM) algorithm, implemented using NS3 software, demonstrates superior performance compared to traditional SVM techniques across multiple metrics, including average energy consumption, average remaining energy, packets received, packet delivery ratio, delay, jitter, throughput, normalized overhead, dropping ratio, and goodput. Simulation results show that E-SVM achieves a 12.5% dropping ratio, 98.459% energy consumption, and an 89.2879% packet delivery ratio, outperforming existing SVM techniques across various network sizes.

## Introduction

Wireless ad-hoc networks are decentralized, self-configuring systems that operate without pre-existing infrastructure, enabling seamless communication in dynamic environments such as disaster recovery, military operations, and IoT systems. However, their lack of centralized control exposes them to severe security vulnerabilities, including packet sniffing, rogue access points, password theft, man-in-the-middle attacks, jamming, wardriving, Bluetooth exploits, and Wired Equivalent Privacy (WEP) breaches. Among these, wormhole and blackhole attacks pose critical threats due to their ability to disrupt routing protocols and compromise data integrity.

In a wormhole attack, malicious nodes create a virtual tunnel to intercept and manipulate network traffic, destabilizing routing paths. Conversely, a blackhole attack involves an unauthorized node masquerading as a legitimate source to capture and discard data intended for the recipient. Both attacks exploit weaknesses in routing protocols, leading to increased packet loss, latency, and energy consumption. Traditional detection methods, which rely on single-layer analysis, suffer from low accuracy, structural rigidity, and slow processing times, rendering them inadequate against evolving threats.

To address these limitations, this study proposes a novel cross-layer defense mechanism that integrates information sharing across the physical, MAC, and network layers. By modeling traffic patterns and analyzing node behavior, the framework identifies anomalies and isolates malicious nodes. The physical layer mitigates channel interference to maintain stable links, the MAC layer monitors transmission failures and bandwidth misuse, and the network layer optimizes routing processes. This collaborative approach enhances threat detection while maintaining protocol independence. The proposed system employs an Enhanced Support Vector Machine (E-SVM) algorithm, outperforming conventional SVM in efficiency, interruption rates, packet delivery ratios, and energy conservation. Simulations using NS3 software demonstrate significant improvements in Quality of Service (QoS) metrics, including reduced false positives and adaptive threat response.

Wireless networks with weak security protocols, such as 802.11 standards, remain vulnerable to intrusions that compromise confidentiality, integrity, and availability. Denial-of-Service (DoS) attacks further degrade performance across multiple layers (Physical, MAC, Network, Transport), highlighting the need for robust, adaptive solutions. This study introduces a conceptual framework for detecting and isolating attacks through linear and nonlinear analysis, addressing heterogeneous network configurations and access point vulnerabilities.

By unifying cross-layer functionalities, this research establishes a resilient defense mechanism that enhances security and operational efficiency in wireless ad-hoc networks. The findings not only overcome the shortcomings of existing methods but also provide a foundation for developing adaptive systems capable of countering sophisticated threats in dynamic environments.

## Related work

The ability of machine learning (ML) to analyze complex patterns in network data has made it a popular tool for detecting wormhole and blackhole attacks. Zhang et al.^1^ introduced a deep learning-based cross-layer system that integrates physical, data link, and network layer components to effectively identify these attacks. Jagadeesan and Parthasarathy^2^ proposed an innovative multi-stage security model consisting of three phases: detection, verification, and prevention. During the detection phase, the model identifies potential Black Hole nodes by analyzing routing behavior and packet delivery ratios. The verification phase utilizes a cross-layer mechanism to confirm suspicious nodes by comparing data from the network and MAC layers. Finally, in the prevention phase, malicious nodes are isolated, and routing tables are updated to avoid them. This approach is efficient and adaptable, as it dynamically responds to network changes and reduces false positives. Additionally, Kumar et al.^3^ developed a hybrid machine learning model that combines supervised and unsupervised learning methods to enhance detection rates in dynamic network environments.

To recognize malicious activity, trust-based systems analyze node behavior at multiple levels. Sharma and Ghosh^4^ introduced a cross-layer trust management system that identifies wormhole and blackhole threats by examining signal strength and transmission behavior. To enhance detection accuracy, Gupta et al.^5^ further developed this approach by incorporating Bayesian inference to actively assess node confidence.

Reddy and Rao^6^ proposed a machine learning-based approach to pinpoint blackhole nodes by detecting abnormalities in signal strength. Adaptive defensive structures have been developed through the use of reinforcement learning (RL). Effectively prevent blackhole and wormhole assaults, Li et al.^7^ presented an RL-based cross-layer design that adapts defense methods based on network conditions. The technique adjusts to changing attack patterns and reduces the number of false positives. The interactions between defenders and attackers were modeled using game theory. Chen et al.^8^ created a game-theoretic cross-layer model that considers security and energy trade-offs when optimizing defense tactics. This approach was further developed by Singh et al.^9^ who utilized multi-agent systems to predict and prevent wormhole attacks. Blockchain technology has been explored to enhance the security of WANETs. Khan and Khan^10^ proposed a blockchain-based cross-layer framework that ensures secure routing by maintaining an immutable ledger of node behavior. This approach mitigates blackhole and wormhole attacks by preventing malicious nodes from participating in routing.

Reshi et al.^11^ propose an innovative defense algorithm to safeguard IoT networks against black hole attacks. Their approach focuses on identifying and isolating malicious nodes by analyzing routing behavior. The algorithm demonstrates effectiveness in maintaining network integrity, making it a valuable contribution to IoT security. Similarly, Yashraj et al.^12^ introduce the security schema RTO-TV against black hole assaults in Mobile Ad Hoc Networks (MANETs). Their trust-value mechanism enhances routing security by evaluating node reliability, offering a robust solution for dynamic ad hoc networks.

Hassan et al.^13^ investigate machine learning and optimization techniques for detecting and mitigating blackhole and greyhole attacks in MANETs. Their work highlights the potential of supervised and unsupervised learning models in identifying anomalous routing behavior. This comprehensive review underscores the importance of adaptive intrusion detection systems in dynamic network environments. Complementing this, Abdallah et al.^14^ propose an anomaly detection approach using Support Vector Machines (SVM) to detect black hole attacks in MANETs. Their method achieves high accuracy in distinguishing malicious nodes, showcasing the efficacy of machine learning in enhancing network security.

Shahid et al.^15^ address the wormhole attack in Wireless Sensor Networks (WSNs) by proposing an energy-optimized cybersecurity approach. Their framework balances security and energy efficiency, ensuring sustainable network operation. This work is particularly relevant for resource-constrained WSNs, where energy drainage attacks pose significant threats. Bhatti et al.^16^ further contribute to this domain by designing a memory-effective data construction arrangement to mitigate energy drainage attacks. Their solution optimizes resource utilization while maintaining robust security, making it a practical advancement for wireless sensor networks.

Lakshmi and Vaishnavi^17^ propose a trusted security approach to detect and isolate routing attacks in MANETs. Their method leverages trust metrics to identify malicious nodes, ensuring secure communication in dynamic environments. This approach aligns with the growing emphasis on trust-based security mechanisms in ad hoc networks. Similarly, Shafi et al.^18^ integrate machine learning and trust-based AODV routing protocols to mitigate flooding and black hole attacks in MANETs. Their hybrid approach enhances routing integrity and network resilience.

May and Atkison^19^ explore the use of propagation delay for wormhole detection in wireless networks. Their technique leverages temporal characteristics to identify malicious links, offering a novel solution to a persistent security challenge. This work complements Prathap Kumar et al.^20^, who propose a routing integrity mechanism to prevent wormhole attacks in vehicular ad hoc networks. Both studies highlight the importance of temporal and spatial analysis in securing wireless communications.

Priyam and Yadav^21^ discuss the challenges, attacks, and countermeasures for securing MANETs in IoT environments. Their work provides a comprehensive overview of the threat landscape and emphasizes the need for adaptive security solutions. This aligns with the broader trend of integrating IoT and MANETs while addressing their unique security requirements. Paul and Sinha^22^ analyze Denial of Service (DoS) attacks in IoT, highlighting their impact on network availability and performance. Their work underscores the importance of proactive defense mechanisms, such as traffic filtering and anomaly detection, in mitigating DoS attacks. This contribution is critical for ensuring the reliability of IoT systems in the face of increasing cyber threats.

Dehury et al.^23^ examine the security issues and challenges in deploying CPS using WSNs. Their work identifies vulnerabilities in CPS architectures and proposes mitigation strategies, emphasizing the need for robust security frameworks in critical infrastructure.

Saleh et al.^24^ provide a complete investigation of safety tests in Wireless Sensor Networks (WSNs) improved by machine learning and deep learning machinery. Their survey highlights the transformative potential of AI-driven solutions in detecting and mitigating sophisticated attacks, paving the way for future research in this domain.

Below, Table 1 compares Machine Learning-Based Detection, Single-Layer Approaches, Heterogeneous Model, SVM, Trust-Based Detection, and Signal Strength and Timing Analysis with the Proposed System E-SVM. The comparison is based on the concepts used, advantages, disadvantages, and system of measurement such as energy consumption, packet delivery ratio, delay, jitter, throughput, and more. A justification for why the proposed system (E-SVM) is superior is also provided.

**Table 1:** Comparative analysis of detection techniques in blockhole and wormhole attacks.

Techniques	Concept used	Advantages	Disadvantages	Performance metrics
Machine learning-based detection	Uses algorithms to detect anomalies or threats based on training data	1. Adaptable to new threats 2. Can handle complex patterns	1. High computational cost 2. Requires large datasets for training	Moderate energy consumption, moderate throughput, high delay, and high overhead
Single-layer approaches	Relies on a single detection mechanism (e.g., rule-based or signature-based)	1. Simple to implement 2. Low computational overhead	1. Limited detection accuracy 2. Not adaptable to new threats	Low energy consumption, low throughput, moderate delay, and low overhead
Heterogeneous model	Combines multiple detection techniques for improved accuracy	1. Robust detection 2. Reduces false positives	1. High energy consumption 2. Increased delay and jitter	High energy consumption, moderate throughput, high delay, and high overhead
SVM (support vector machine)	Uses supervised learning for classification and regression tasks	1. Effective for high-dimensional data 2. Good generalization ability	1. Computationally intensive 2. Requires careful parameter tuning	Moderate energy consumption, moderate throughput, moderate delay, and moderate overhead
Trust-based detection	Evaluates node trustworthiness to detect malicious activities	1. Reduces false positives 2. Effective in collaborative networks	1. High overhead 2. May drop legitimate packets	High energy consumption, moderate throughput, high delay, and high overhead
Signal strength and timing analysis	Uses physical layer information (e.g., RSSI, time delays) for detection	1. Improves localization accuracy 2. Effective for physical layer attacks	1. Sensitive to environmental changes 2. High computational cost	High energy consumption, moderate throughput, high delay, and high overhead
Proposed system E-SVM	Enhanced SVM with optimization for energy efficiency and detection accuracy	1. Higher detection accuracy 2. Energy-efficient 3. Scalable	1. Requires initial training 2. Slightly higher computational complexity during training	Low energy consumption, high throughput, low delay, low overhead, and high goodput

## Methodology

**Fig. 1:**
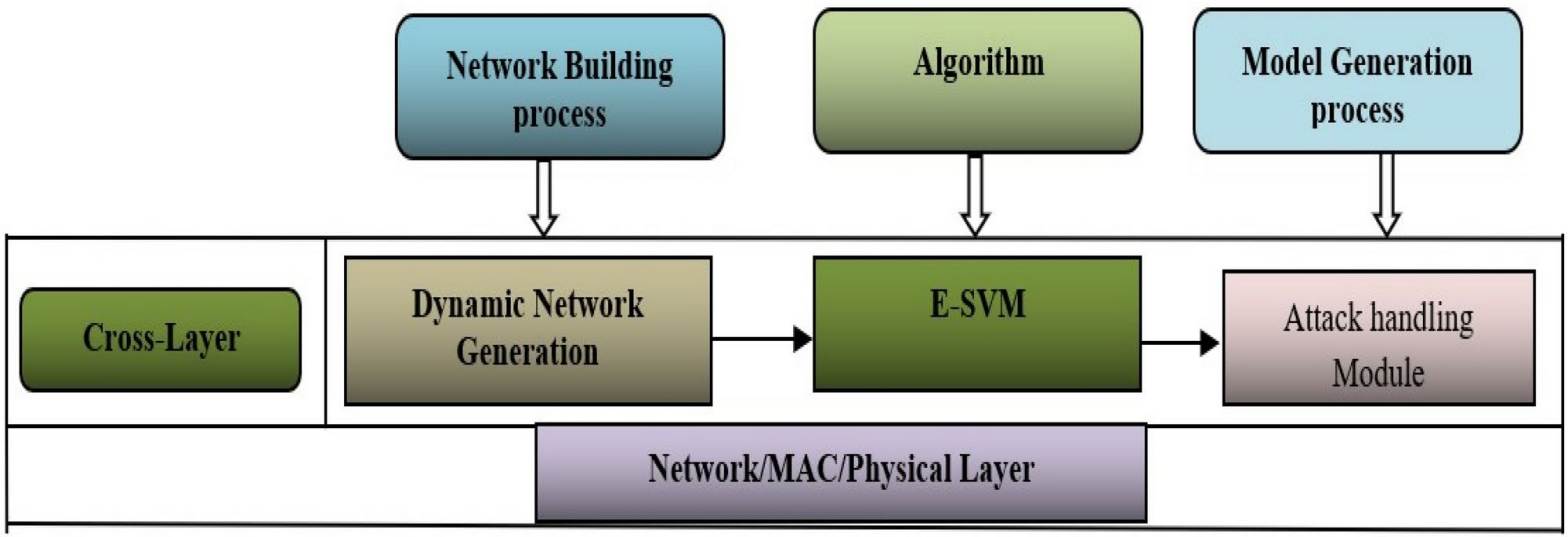
Proposed cross-layer attacking handling approach.

Wireless security can be provided as a part of protocols but still suffers when network traffic is high. The paper extends the SVM algorithm by enhancing the same for handling the blackhole and wormhole attacks. The proposed cross-layer-based E-SVM for handling attack architecture is shown in Fig. 1. The Enhanced Support Vector Machine (E-SVM) is an advanced variant of the traditional Support Vector Machine (SVM) algorithm. It is designed to improve performance by incorporating Radial Basis Function (RBF) kernels to handle non-linear data relationships and Particle Swarm Optimization (PSO) for optimal feature selection. This enhancement leads to increased accuracy and efficiency. In the context of wireless ad-hoc networks, which are decentralized and consist of wireless devices communicating directly with each other, E-SVM plays a crucial role in detecting attacks such as blackhole attacks and wormhole attacks. E-SVM enhances detection accuracy by effectively identifying anomalies, optimizes the detection process for faster and more reliable performance, and adapts to various network conditions and types of attacks, providing a robust defense mechanism.

### A case of linear classification

Suppose we have empirical data points *(x*_1_*, y*_1_*) (x*_2_*, y*_2_*) . . . (x*_m_*, y*_m_*)* ∈ *R* × {− 1, + 1}. In linear classification, the Support Vector Machine (SVM) algorithm computes a hyperplane that optimally separates samples from two classes. The function *f* is linear in *x*_*i*_ with the following general form:

1 $$f\left( {W,xi,b} \right) = \left\langle {w,xi} \right\rangle + b$$

While there are infinitely many hyperplanes that can separate the data, only one is optimal the hyperplane that maximizes the margin. This optimal hyperplane satisfies the condition is

2 $$gi = yif \left( {w, xi ,b} \right){-} 1\quad {\text{where}}\quad gi \ge 0\quad for\quad i = 1 \ldots m$$

To set up the optimization problem of finding a maximal margin hyperplane is to observe that for two points x + and x− that lie nearest to it, it is true that

3 $$\begin{gathered} \left\langle {w, x^{ + } } \right\rangle + b = + 1 \hfill \\ \left\langle {w, x^{ - } } \right\rangle + b = - 1 \hfill \\ \left\langle {w, (x^{ + } - x^{ - } )} \right\rangle = 2 \hfill \\ \left\langle {\frac{w}{\left\| w \right\|},\left( {x^{ + } - x^{ - } } \right)} \right\rangle = \frac{2}{\left\| w \right\|} \hfill \\ \end{gathered}$$

Hence, the margin is inversely proportional to the norm of w. Therefore, we need to minimize the norm of w subject to the given constraints, that is $Minim ize:$

4 $${\text{F}} = \frac{{\left\| w \right\|^{2} }}{2}$$

5 $$subject\;to:\quad g_{i} \ge 0 \forall i$$

This equation can be solved by constructing a Lagrangian function:

6 $$F_{P} = F + \lambda_{i } g_{i} \quad {\text{Where}}\quad \lambda_{i} \ge 0$$

the Lagrange multipliers are

7 $$\begin{gathered} = \frac{{\left\| w \right\|^{2} }}{2} - \lambda_{i} \left( {y_{i} f\left( {w, x_{i} ,b} \right){-} 1} \right) \hfill \\ = \frac{{\left\| w \right\|^{2} }}{2} - \mathop \sum \limits_{i = 1}^{m} \lambda_{i} \left( {y_{i} \left( {\left\langle {w, x_{i} } \right\rangle + b} \right){-} 1} \right) \hfill \\ = \frac{{\left\| w \right\|^{2} }}{2} - \mathop \sum \limits_{i = 1}^{m} \lambda_{i} y_{i} wx_{i} - \mathop \sum \limits_{i = 1}^{m} \lambda_{i} y_{i} b + \mathop \sum \limits_{i = 1}^{m} \lambda_{i} \hfill \\ \end{gathered}$$

The Lagrange multiplier coefficient, denoted as λ_i_, plays a crucial role in determining the position of the support vectors. These support vectors are the nearest points that influence the optimal conditions when transforming the primal problem into its corresponding dual Lagrangian.

8 $$\frac{{\partial F_{P} }}{\partial w} = 0 \Rightarrow w = \mathop \sum \limits_{i = 1}^{m} \lambda_{i} y_{i} x_{i}$$

9 $$\frac{{\partial F_{P} }}{\partial b} = 0 \Rightarrow \mathop \sum \limits_{i = 1}^{m} \lambda_{i} y_{i} = 0$$

The result is a quadratic programming problem with linear constraints

10 $$F_{D} = \mathop \sum \limits_{i = 1}^{m} \lambda_{i} - \frac{1}{2}\mathop \sum \limits_{i,j} \lambda_{i} \lambda_{j} y_{i} y_{j} x_{i} , x_{j} ,\mathop \sum \limits_{i = 1}^{m} \lambda_{i} y_{i} = 0$$

11 $$\begin{gathered} y_{i} \left( {\left\langle {w, x_{i} } \right\rangle + b} \right){-} 1 \ge 0\quad i = 0,1, \ldots m \hfill \\ \lambda_{i} \ge 0\quad \forall i \hfill \\ \lambda_{i} \left( {y_{i} f\left( {w, x_{i} ,b} \right) {-}1} \right) = 0\quad \forall i \hfill \\ \end{gathered}$$

So, the weight hyperplane vector $w = \mathop \sum \limits_{i = 1}^{m} \lambda_{i} y_{i} x_{i}$ and the offset $b = 1 - wx_{i}$, where $x_{i}$ is support vector of the known class (here its class is 1), The resulting solution has the property that in fact, often most of the coefficients $\lambda_{i}$ are equal to zero. The only positive coefficients correspond to the points that lie closest to the hyperplane, and for this reason, such points go under the name of support vectors. The final decision function:

12 $$f\left( {w, x,b} \right) = \left\langle {w,x} \right\rangle + b = \mathop \sum \limits_{i = 1}^{m} \lambda_{i} y_{i} \left\langle {x_{i} , x} \right\rangle + b$$

where the index *i* runs only on the support vectors. In other words, if all data points other than the support vectors were removed, the algorithm would find the same solution.

### A case of nonlinear classification

The separator hyperplane from the preceding section is no longer relevant in scenarios involving nonlinear classification. As such, a non-linear Support Vector Machine (SVM) must be used. The underlying idea of this method is to find a high-dimensional space (usually called a Hilbert space, abbreviated as H) where the examples’ projections become separable linearly. We substitute the simple scalar product in this modified space, which corresponds to the original space of observations, with a kernel function.

We suppose:

13 $$\varphi : R^{P} \to H \quad {\text{defined}}\;{\text{by}}\left( {x_{i} } \right) = x_{i}$$

By replacing the scalar product $\varphi \left( {x_{i} } \right).\varphi \left( {x_{j} } \right)$ by a kernel function $K\left( {x_{i} , x_{j} } \right)$, the problem of optimization becomes:

Maximize:

14 $$F_{D} = \mathop \sum \limits_{i = 1}^{m} \lambda_{i} - \frac{1}{2}\mathop \sum \limits_{i,j} \lambda_{i} \lambda_{j} y_{i} y_{j} K(x_{i, } x_{j} )$$

Subject to

15 $$0 \le \lambda_{i} \ge C\quad {\text{and}}\;\mathop \sum \limits_{i = 1}^{m} \lambda_{i} y_{i} = 0$$

where *C* is the tolerance constant. The decision function evaluated on the support vector $x_{i}$ is:

16 $$f\left( {w, x,b} \right) = \mathop \sum \limits_{i = 1}^{m} \lambda_{i} y_{i } e^{{ - \frac{1}{ 2}\left( {\frac{{\left\| {x_{i } ,x} \right\|}}{\sigma }} \right)^{2} }} + b$$

The Enhanced Support Vector Machine (E-SVM) algorithm utilizes linear classification when the data is linearly separable. Network traffic features are extracted and represented in vector form. A linear kernel function calculates the decision boundary, and the optimal hyperplane is determined by maximizing the margin between classes using the standard SVM objective function. New data points are then classified based on their position relative to the hyperplane. For more complex data patterns that are not linearly separable, E-SVM employs a nonlinear kernel. Network traffic features are extracted similarly, but a nonlinear kernel function, such as a polynomial or radial basis function (RBF), is utilized to map the input features into a higher-dimensional space.

In the proposed work section, we will discuss how the proposed E-SVM algorithm has been applied to detect blackhole and wormhole attacks in wireless ad-hoc networks.

## Proposed work

The paper extends the SVM approach for Blackhole and Wormhole attacks in wireless ad-hoc networks. The performance of the algorithm is detailed in the following sections, and the different components in the proposed model are described.

- *Wormhole attacks Device* The devices create the virtual tunnel to capture and discard the data packets.
- *Blackhole attacks Devices* It generates a fake route reply message that announces the falsified shortest route to connect to the destination.
- *Source node* Nodes that involve generating the data packets to intended destinations.
- *Intermediate nodes* Nodes receive and forward the data packet using the constructed route.
- *Destination devices* Nodes accept the data packet from the source node.
- *Attack detection system* The training and testing system used to differentiate the nodes behaviours based on the data transmission using the SVM classifier.

### The E-SVM algorithm working mechanism

The working mechanism of the Enhanced Support Vector Machine (E-SVM) algorithm starts with randomly deploying wireless devices in a specific topological area within a designated communication range. These devices are organized based on graph properties to ensure all nodes are connected. Neighborhood communication is established using beacon messages, which are broadcast periodically over the wireless medium. Receiver devices update their neighbor tables with the transmitter’s information and the expiration time of links. To maintain accuracy, each device uses a neighbor timer to remove expired entries.

When a source node generates a data packet at the application layer, it signals the network layer through the transport layer, which then looks for a routing table entry to the intended destination. Initially, if no route is found in the neighbor table, the source node initiates a route discovery process by broadcasting a route request message across the network. Neighboring devices verify the message for freshness and routing loops, discarding duplicates. If a device is a destination, it creates a route reply message using the reverse path of the request message; otherwise, the message is rebroadcast until it reaches the destination. The destination node sends the route reply message back to the source node through the established path. Upon receiving it, the source node begins transmitting data packets along this path.

However, during route discovery, blackhole nodes may send falsified route replies to capture data packets, and wormhole nodes may create virtual tunnels to forward packets between themselves, forming a deceptive shortest path connecting the source and destination nodes. Identifying these malicious devices is accomplished through behavioral pattern classification within the SVM classifier. The algorithm consists of two phases: the Setup Phase and the Detection and Isolation Phase.

### Setup phase

The setup phase involves route construction and data transmission processes carried out by wireless devices in the network. Malicious devices may capture and discard data to degrade the Quality of Service (QoS) in communication. To address this, the detection system within the devices performs a data collection process that identifies key metrics such as the number of route requests, replies, data forwarding instances, and packet drop counts. Based on these input parameters collected from each node in the network, the detection process is initiated.

### Detection and isolation phase

To detect behavioral variations in devices, it is essential to first establish a baseline of normal behavior patterns. Key indicators of node behavior include metrics such as the number of route requests generated and forwarded, route replies generated and forwarded, data packets received and forwarded, and packet drop counts. During the route discovery process, the number of route requests and replies both generated and forwarded is calculated for each device in the neighborhood. In the data transmission phase, the number of data packets received and forwarded, along with the packet drop count, is recorded for each participating device. These parameters are used to classify malicious devices based on deviations from normal behavior. The Support Vector Machine (SVM) classification process begins by training the network using the behavioral patterns of devices. Each parameter is treated as a dimension in the classification training input. The mean value for each dimension is calculated by summing the values and dividing by the number of entries. The variance is computed by summing the absolute differences between each value and the mean value. The standard deviation is then derived as the square root of the variance. The lower and upper bounds of the trained input dimension are determined by subtracting and adding the standard deviation to the mean, respectively.

For the test input, the identified parameters of each node are compared against the trained input sequence. These parameters are organized into multiple dimensions. In each dimension, the average values of the trained input and the current input dimension of the node being tested are structured into a permutation set. Within this set, the mean value, sum of absolute differences, variance, and standard deviation are calculated. The weighted factor for the SVM classification is derived using the inverse negative exponential distribution of the parameter set’s average. The Permutation Importance (PI) value for both the trained and test values in the permutation set is calculated by taking the square root of the sum of the squares of the permutation values. If this calculated value exceeds a certain threshold, a specific sum is added to the negative sum of the permutation output and the absolute difference between the permutation input and output. This permutation computation is repeated for both the trained sequence and the test value. The SVM classification value is the sum of the product of the weighted factor with the permutation values of the trained sequence and the test input in each dimension. A negative classification value indicates a behavioral deviation in the wireless nodes, signalling potential malicious behavior. The enhanced SVM classification process differentiates patterns into linear and non-linear classifications. Linear classification occurs when the test input parameter falls within the lower and upper bounds of the trained sequence, while non-linear classification occurs when it does not. In linear classification, both the trained sequence bounds and the test input are arranged into a 2 × 2 matrix, from which eigenvalues are calculated using the quadratic equation. The positive root of the equation is used as the eigenvalue, and the permutation value of both the trained sequence and test input is computed as the square root of their product sum. The weight factor is then derived from the sum of the product of the eigenvalue, permutation value, and test input value, with the coefficient ‘b’ being the difference of this value from one. These calculations determine the final decision value of the test input.

For non-linear classification, the permutation value is computed using a negative exponential with the Gaussian distribution function, and the weighted coefficient is added to form the final decision value. This value helps distinguish normal behavior from malicious behavior in wireless nodes. Nodes exhibiting deviated behavior are identified as malicious and subsequently excluded from data transmission.

**Algorithm 1:**
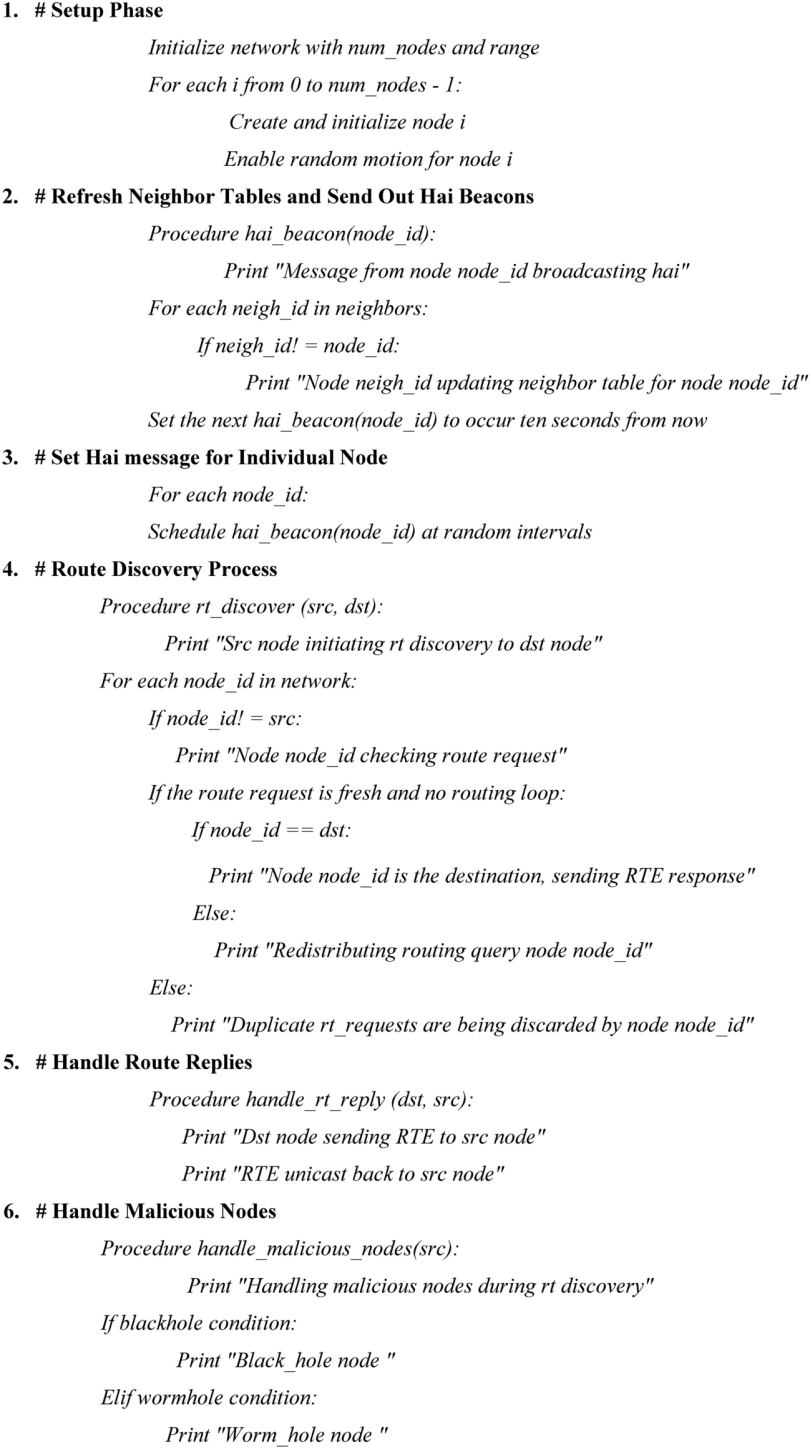
The E-SVM Algorithm for detecting blackhole and wormhole attacks

Algorithm 1 initializes a wireless ad-hoc network with nodes configured for random motion, dynamically refreshing neighbor tables using hai beacons broadcast at randomized intervals. During the route discovery phase, nodes check route requests, eliminate duplicates to avoid redundancy, and respond to valid route replies to establish communication paths. To address security threats, the algorithm detects blackhole attacks by identifying falsified route replies injected into the network and mitigates wormhole attacks by monitoring for maliciously generated virtual tunnels and fake shortest routes. By continuously updating node states and managing communication integrity in real time, the framework ensures dynamic, secure, and efficient network operations.

## Results and discussion

The simulations are carried out in NS3, and the runtime environment is set up with the following parameters.

Table 2 shows the network parameters and setup configuration. The number of nodes is generalized for 50 to 250 nodes traffic for understanding the behaviour of the proposed algorithm. The entire simulations have been carried out for SVM and E-SVM and the results are presented.

**Table 2:** Network parameters and its setup configuration.

Parameter	Value
No of nodes	50 to 250
Mac type	802.11
Queue type	Priority queue
Coverage area	250
Topology area	1000 × 1000
Antenna type	Omnidirectional
Simulation time	250 s
Buffer length	50
Connection type	UDP
Traffic	CBR
Packet size	2000bytes
Packet generation interval	0.1 0.2 0.3 0.4 0.5

**Fig. 2:**
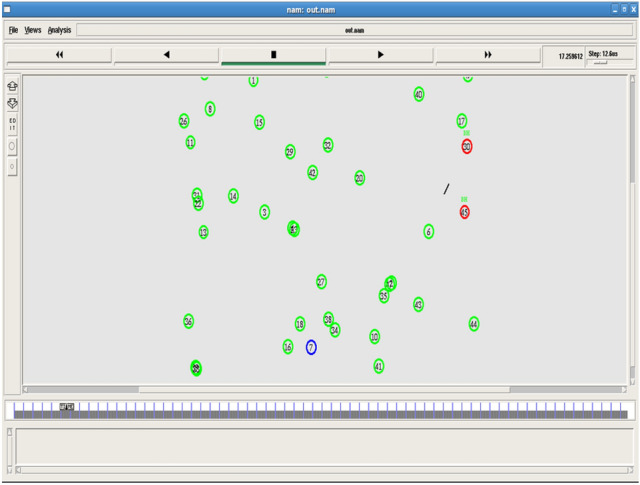
Node deployment.

**Fig. 3:**
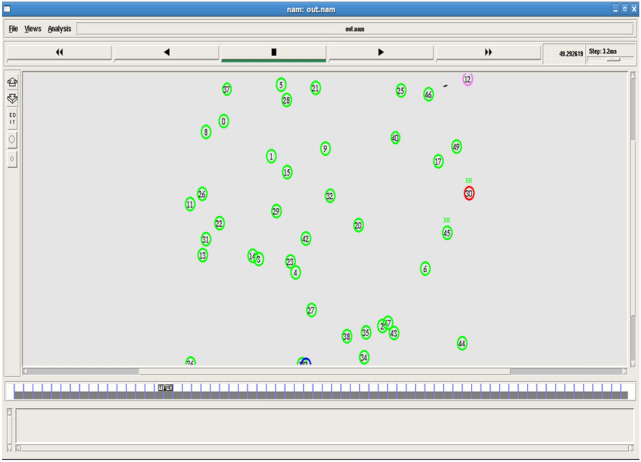
Beacon message communication.

Figures 2 and 3 shows the node deployment and beacon message communication. This sets the initial network for analysis.

**Fig. 4:**
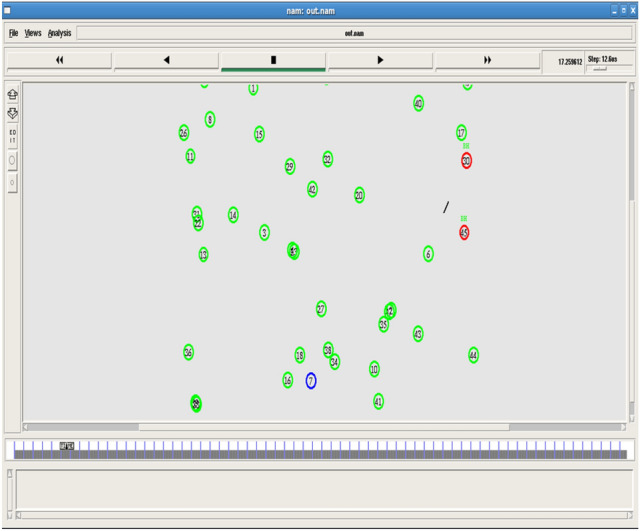
Route discovery process.

**Fig. 5:**
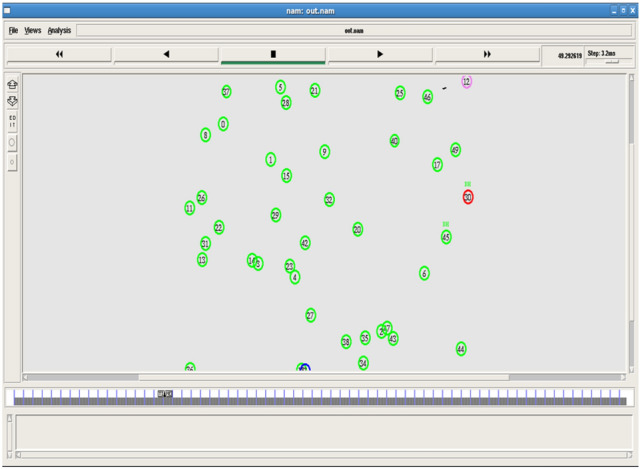
Attacker capturing packets.

**Fig. 6:**
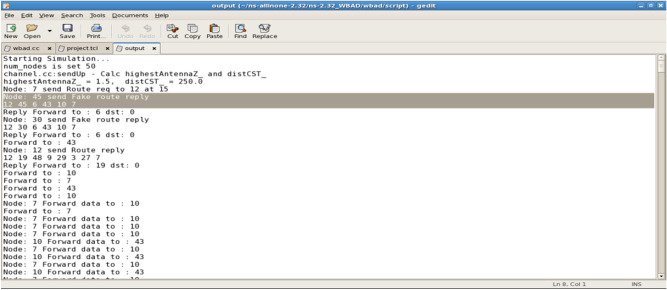
Forwarding datathrough Alternate_Route.

In Fig. 4 the route discovery process is illustrated, while Fig. 5 depicts an attacker capturing packets. Both processes are managed using the SVM algorithm and the E-SVM algorithm. Figure 6 showcases Node 45 sending a fake route reply as part of the attack scenario.

**Fig. 7:**
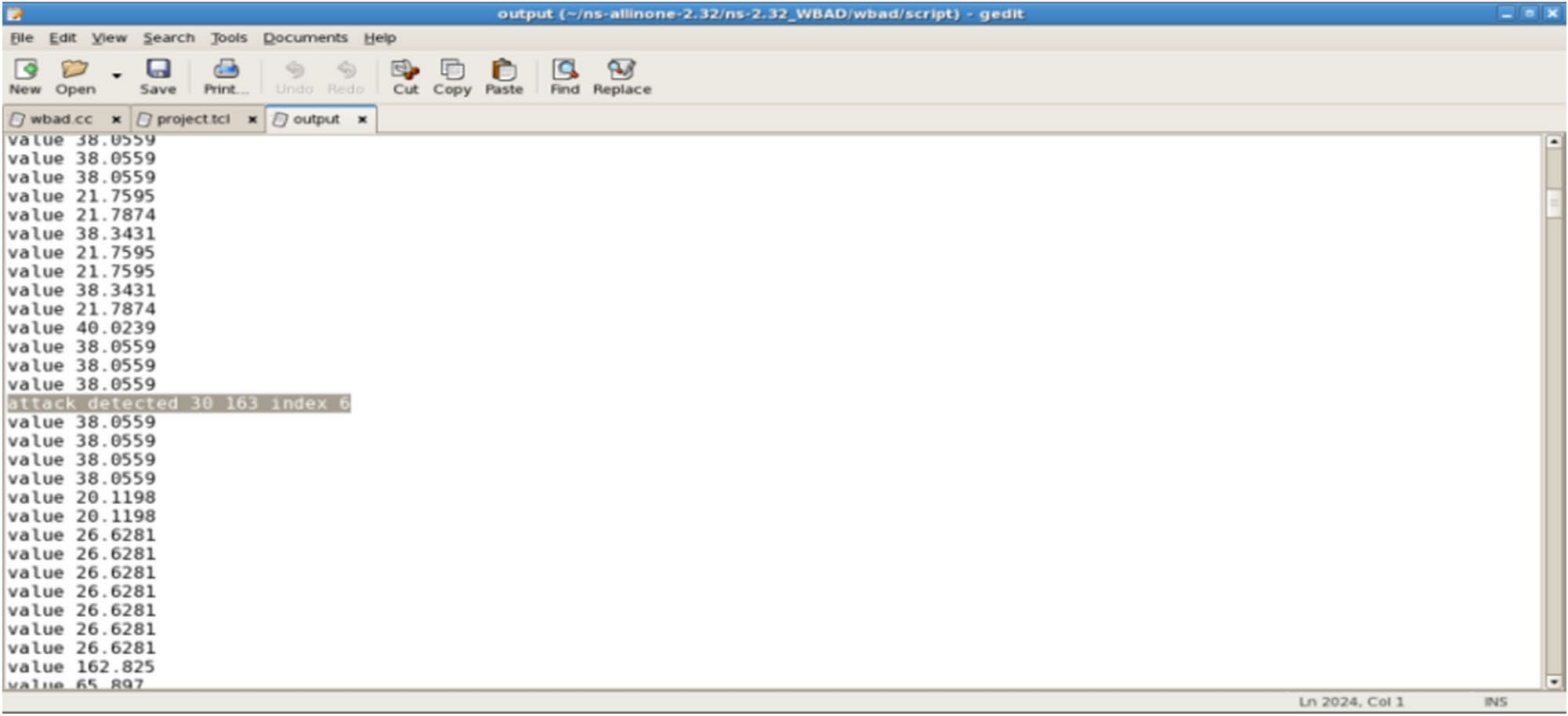
Attacker_detected_30_by_Node_6_Packet_drop_count_163.

The attack is detected by the SVM module, as illustrated in Fig. 7. In this scenario, Node 6 identifies the attacker, labeled as Attacker 30, resulting in a packet drop count of 163.

**Fig. 8:**
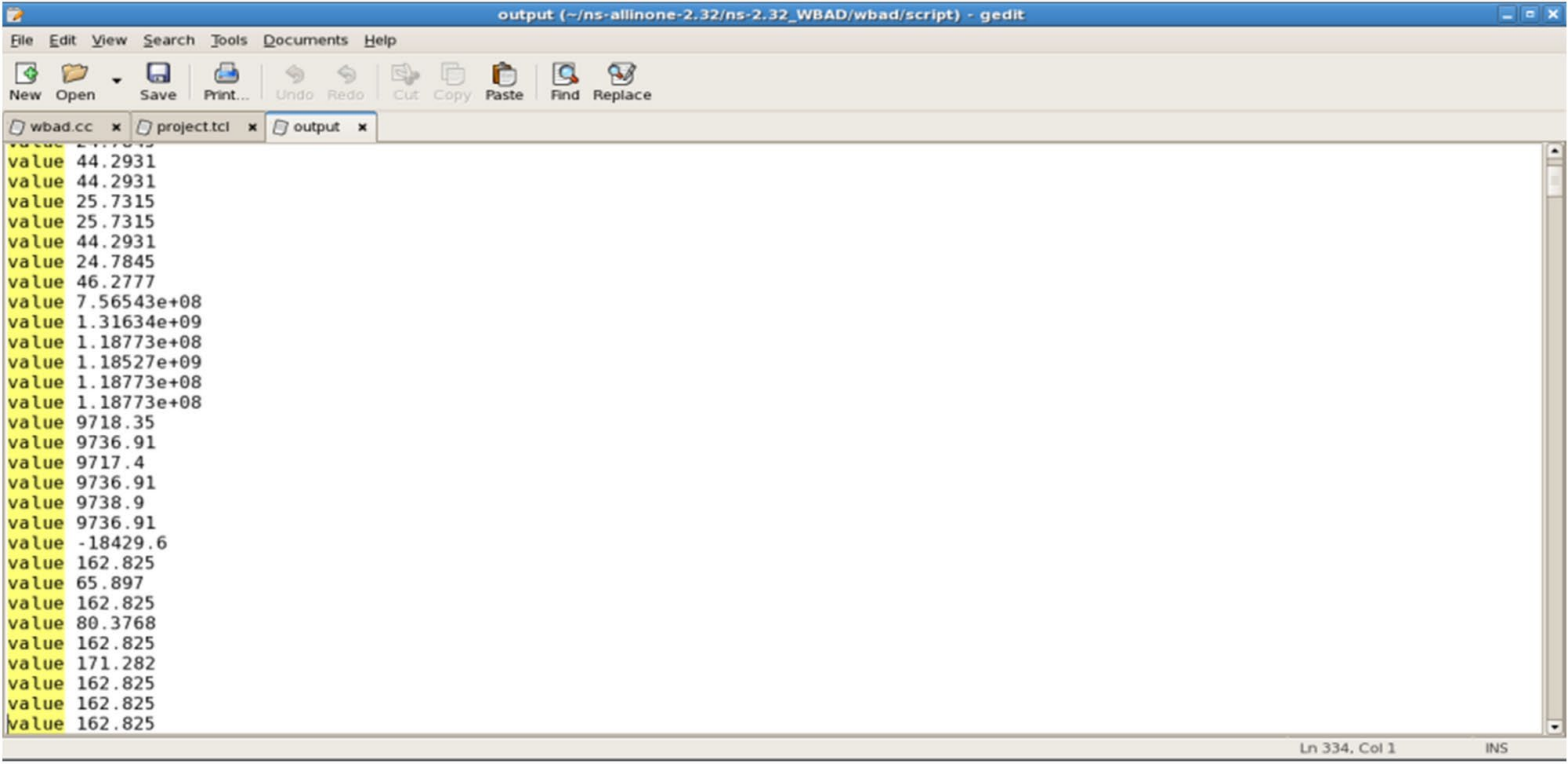
The SVM_Classifier Value for the transmissions.

The SVM algorithm classifies and presents its values, as illustrated in Fig. 8. This figure displays the transmission values provided by the SVM classifier.

**Fig. 9:**
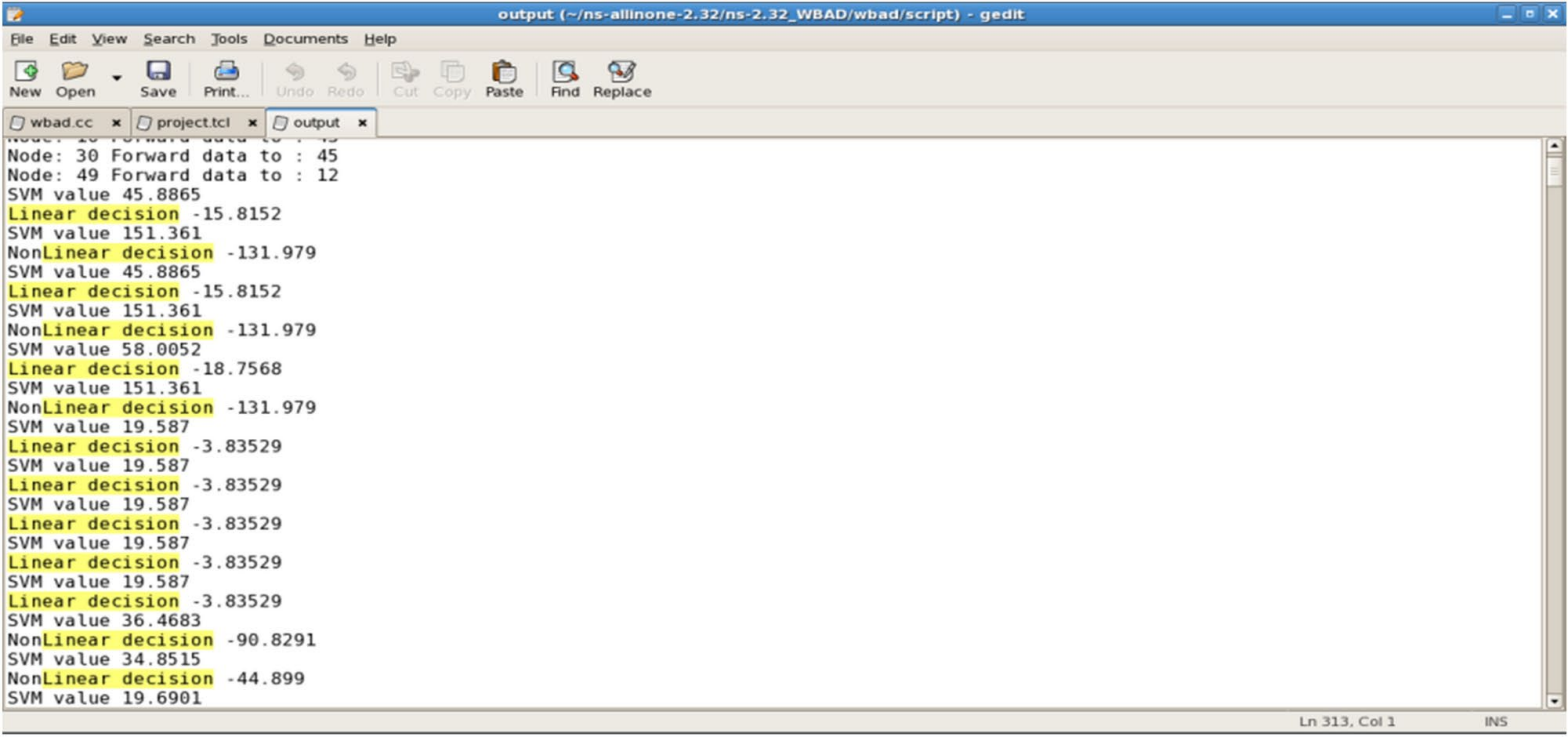
A Linear nonlinear decision.

The linear and nonlinear decision cases are presented for each of the transmissions, as illustrated in Fig. 9.

The remaining graphs display the performance parameters for both SVM and E-SVM, including average consumed energy, average remaining energy, packets received, packet delivery ratio, delay, jitter, throughput, normalized overhead, dropping ratio, and goodput.

**Fig. 10:**
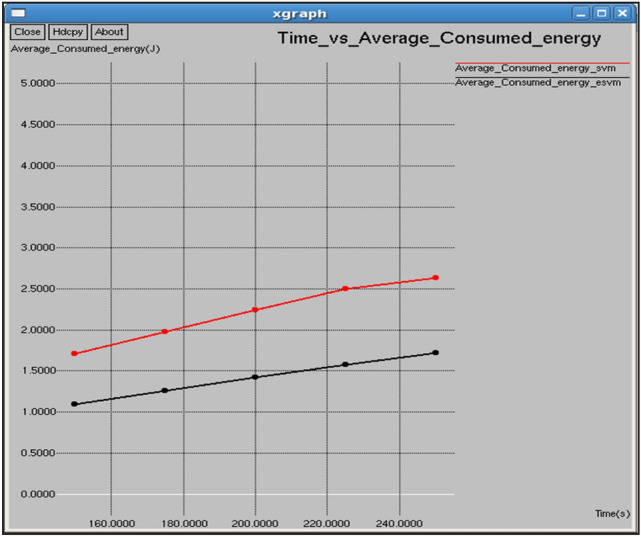
Time vs average energy.

**Fig. 11:**
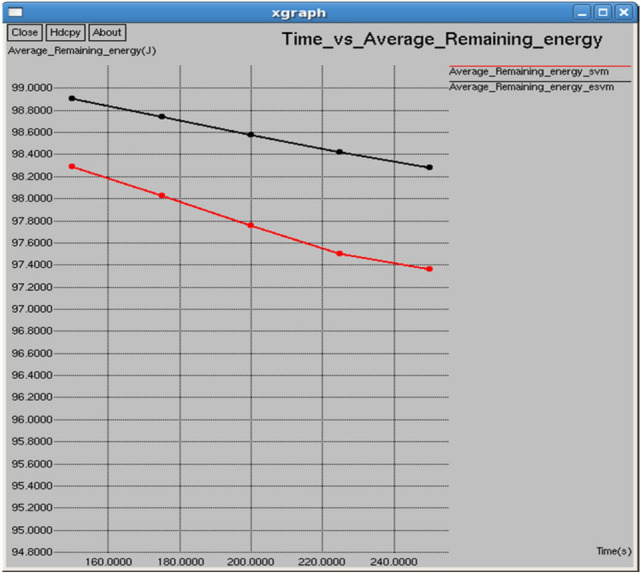
Time vs average remaining energy.

Figures 10 and 11 show that the E-SVM maintains a higher average remaining energy and lower average consumed energy compared to the standard SVM during the observed time intervals. At 0.1 s, E-SVM has an average remaining energy of 98.9055 J and consumed energy of 1.7081 J, outperforming SVM, which has 97.363 J remaining energy and 2.636 J consumed energy. The E-SVM method is more energy-efficient and effective in conserving energy than the SVM approach.

**Fig. 12:**
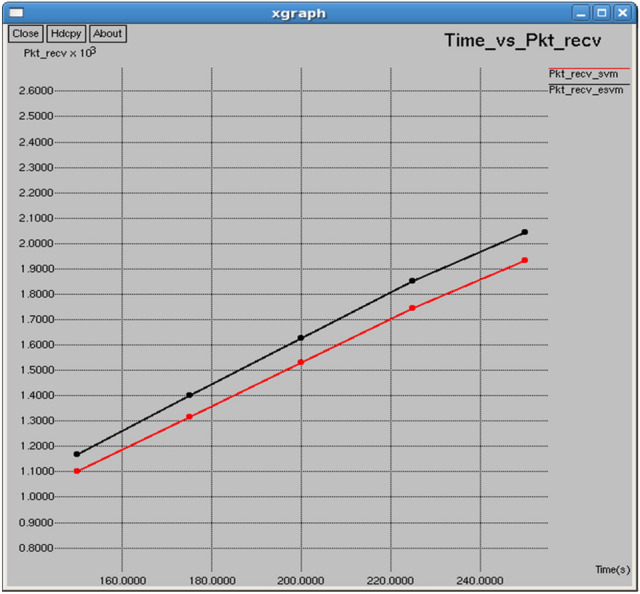
Time vs packets received.

**Fig. 13:**
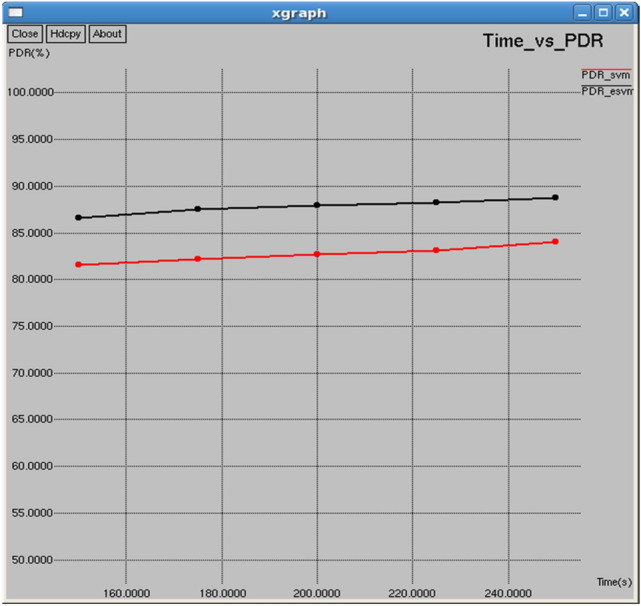
Time vs PDR.

The comparison in Figs. 12 and 13 highlights the superior performance of E-SVM over SVM in terms of packets received and Packet Delivery Ratio (PDR). At 250 s, E-SVM receives 2043 packets with a PDR of 88.7875%, significantly surpassing SVM’s 1,933 packets and 84.007% PDR. These results demonstrate that E-SVM consistently delivers more packets successfully and maintains a higher PDR, showcasing its superior reliability and efficiency in network communication.

**Fig. 14:**
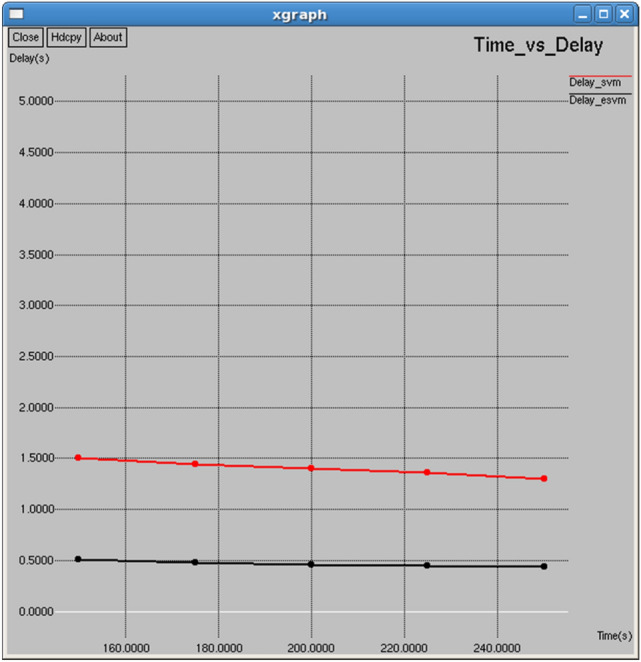
Time vs delay.

**Fig. 15:**
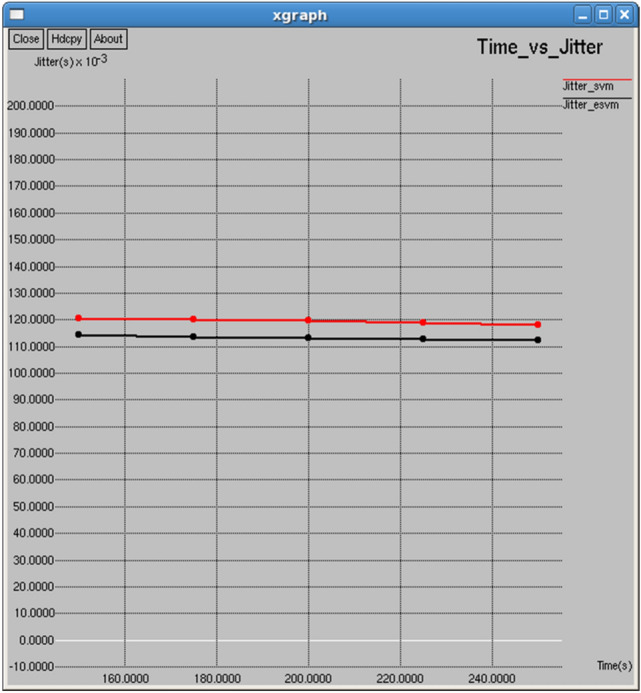
Time vs jitter.

Figures 14 and 15 demonstrate that the E-SVM outperforms the standard SVM in terms of both jitter and delay across different time intervals. For example, at 250 s, the E-SVM achieves a jitter of 0.112224 s slightly lower than the SVM of 0.118247 s, and a delay of 0.438759 s, significantly surpassing the SVM delay of 1.30109 s.

**Fig. 16:**
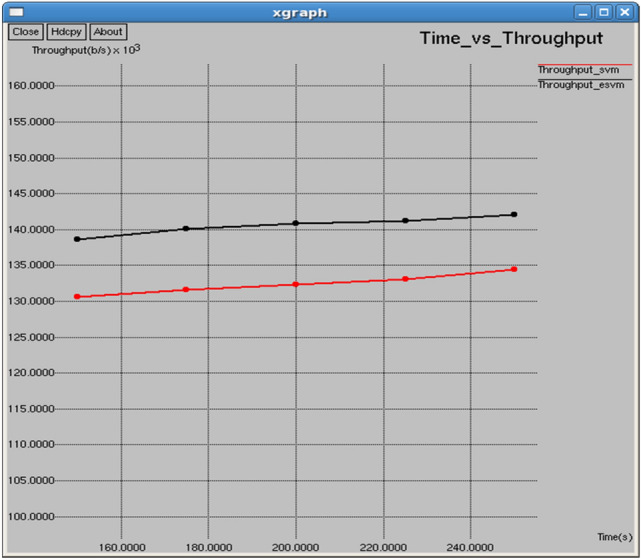
Time vs throughput.

**Fig. 17:**
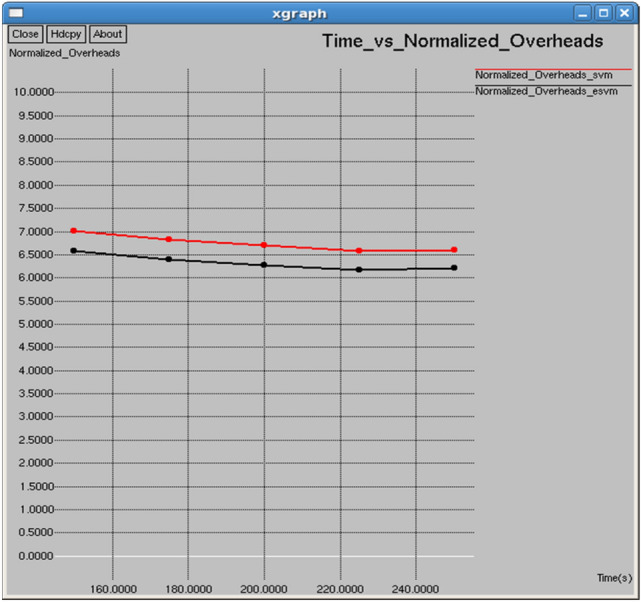
Time vs normalized overheads.

Figures 16 and 17 show that E-SVM achieves higher throughput and lower Normalized Routing Overhead (NRO) compared to SVM across different time intervals. At 200 s, E-SVM reaches a throughput of 140,790 packets/s and an NRO of 6.26245, outperforming SVM’s throughput of 132,309 packets/s and NRO of 6.69588.

**Fig. 18:**
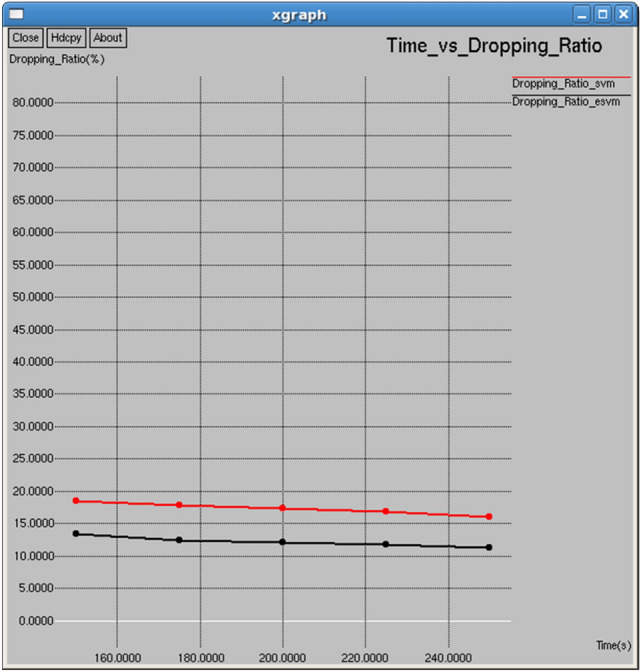
Time vs dropping ratio.

**Fig. 19:**
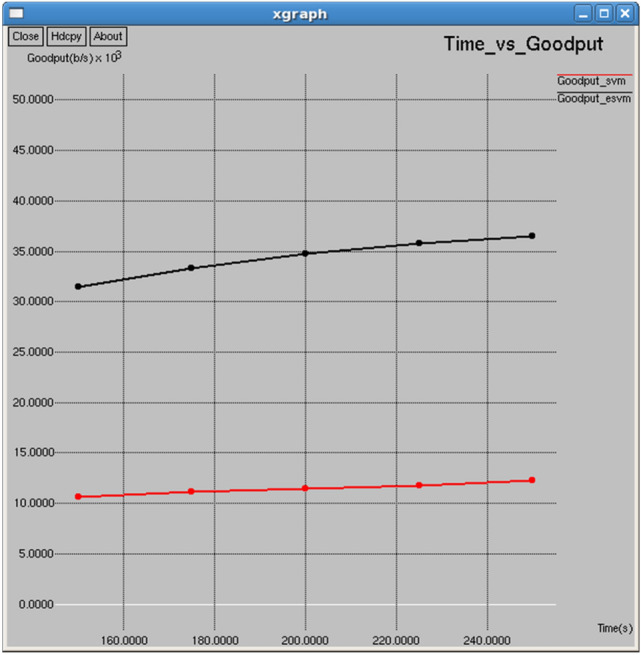
Time vs Goodput.

Figures 18 and 19 show that E-SVM achieves lower dropping ratios and higher goodput compared to SVM across various time intervals. For example, at 175 s, E-SVM achieves a dropping ratio of 12.5% and a goodput of 33,289 b/s, outperforming SVM’s dropping ratio of 17.8125% and a good put of 11,107.3 b/s. This demonstrates E-SVM’s greater ability to minimize packet loss and improve data transmission efficiency.

**Fig. 20:**
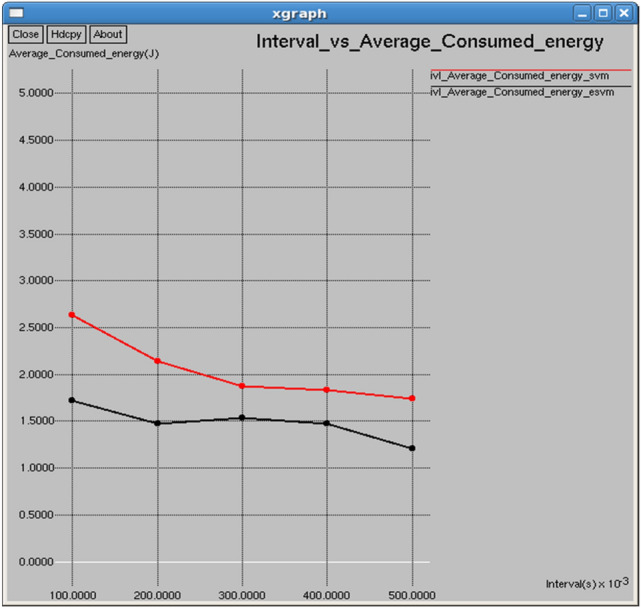
Interval vs average consumed energy.

**Fig. 21:**
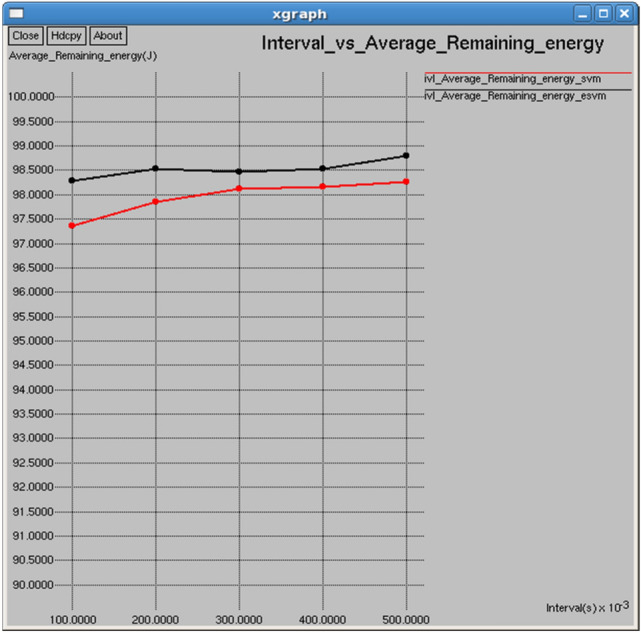
Interval vs average remaining energy.

Figures 20 and 21 demonstrate that E-SVM maintains a higher average remaining energy and lower average consumed energy compared to SVM across various time intervals. At 0.3 s, E-SVM retains 98.4599 J of energy and consumes 1.42167 J, outperforming SVM, which retains 98.1279 J and consumes 2.24201 J.

**Fig. 22:**
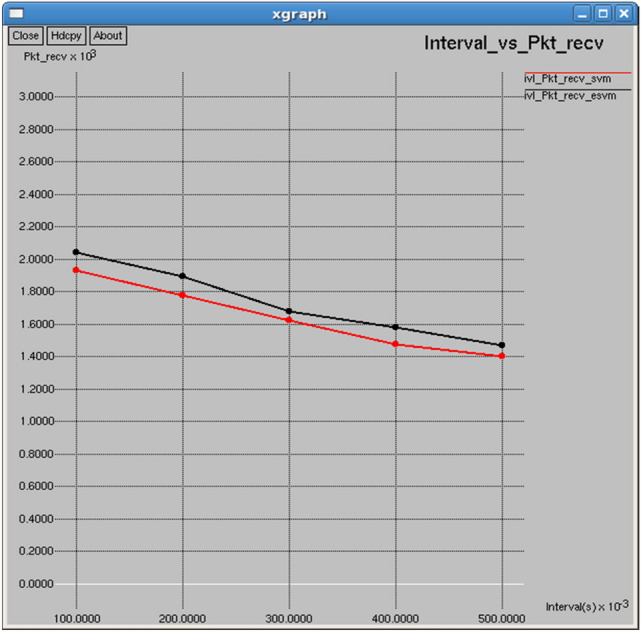
Interval vs packets received.

**Fig. 23:**
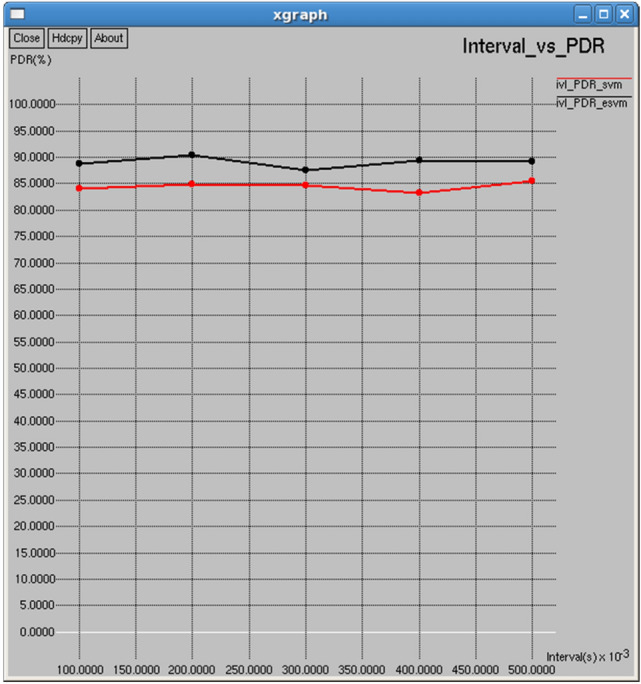
Interval vs packet delivery ratio.

Figures 22 and 23 reveal that E-SVM consistently outperforms SVM in both packets received and packet delivery ratio (PDR) over various intervals. For example, at 0.5 s, E-SVM receives 1,467 packets with a Packet Delivery Ratio (PDR) of 89.2879%, outperforming SVM, which receives 1,404 packets with a PDR of 85.4534%, demonstrating its superior reliability and efficiency in packet delivery**.**

**Fig. 24:**
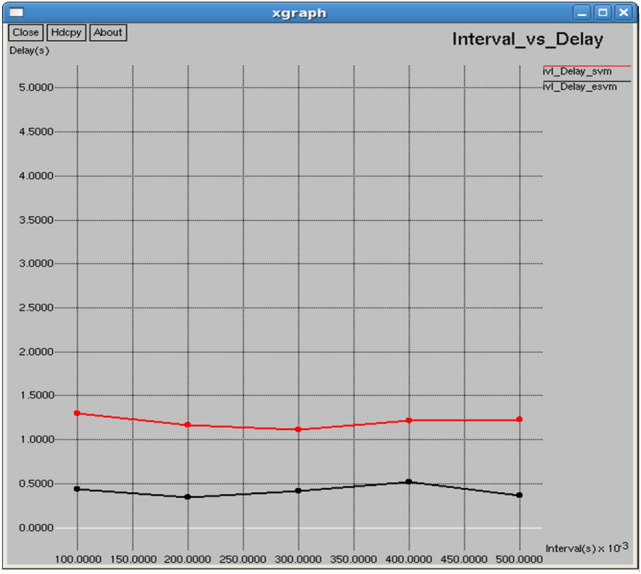
Interval vs delay.

**Fig. 25:**
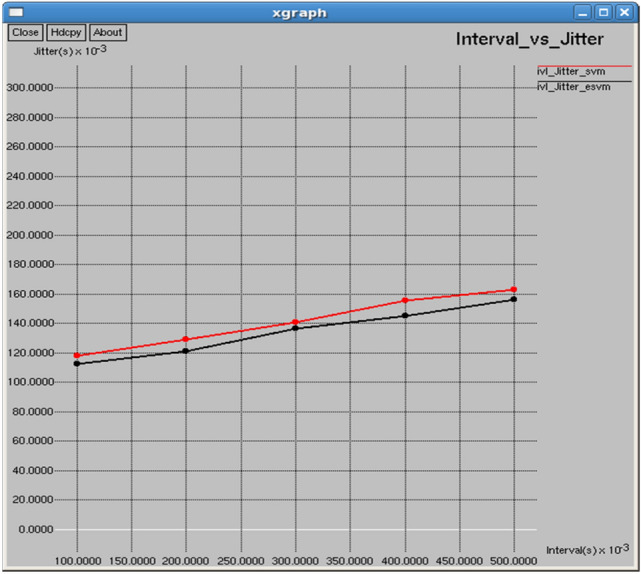
Interval vs jitter.

Figures 24 and 25 demonstrate that E-SVM achieves lower delay and jitter values compared to SVM across different time intervals. For example, at the 0.3-s interval, E-SVM has a delay of 0.414718 s and jitter of 1.54014 s, outperforming SVM’s delay of 1.11558 s and jitter of 1.87212 s.

**Fig. 26:**
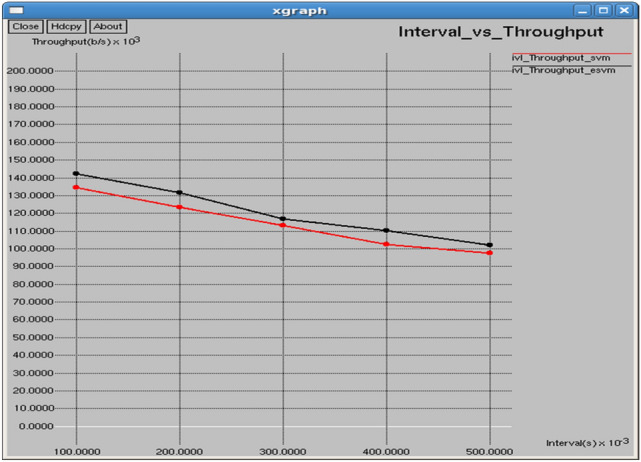
Interval vs throughput.

**Fig. 27:**
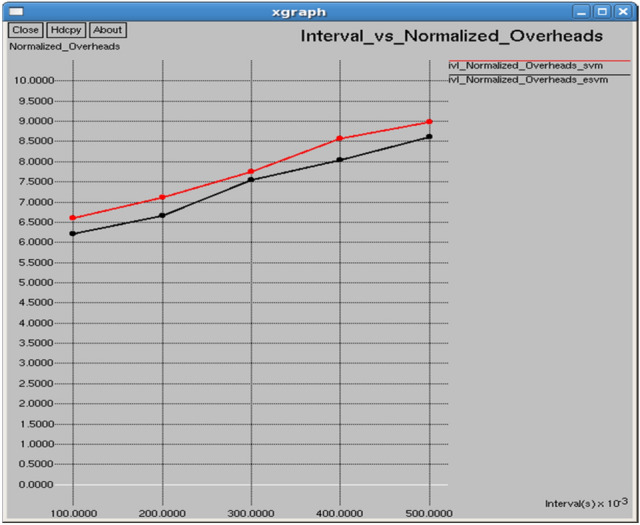
Interval vs normalized overheads.

Figures 26 and 27 illustrate that E-SVM attains higher throughput and lower Normalized Routing Overhead (NRO) compared to SVM across several time intervals. For example, at 0.3 s, E-SVM reaches a throughput of 116,910 b/s and an NRO of 7.54226, outperforming SVM’s throughput of 113,013 b/s and NRO of 7.74631.

**Fig. 28:**
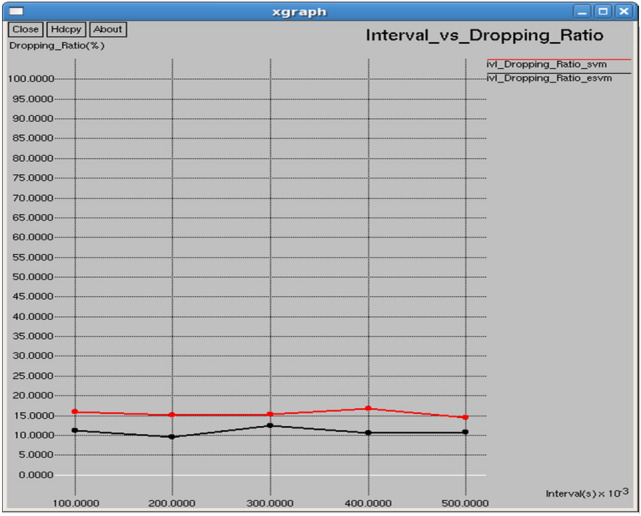
Interval vs dropping ratio.

**Fig. 29:**
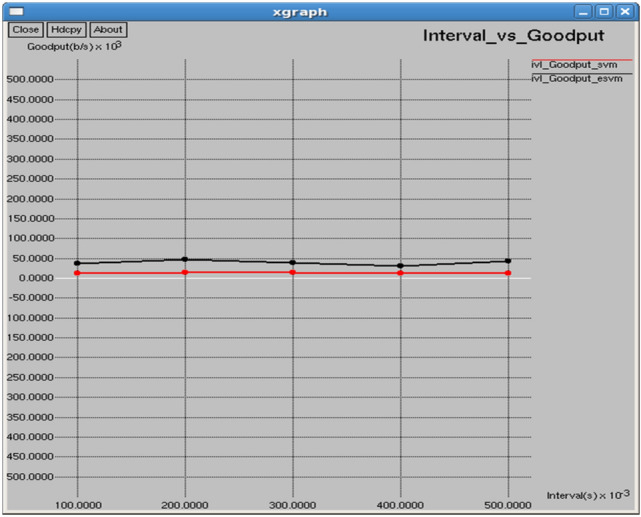
Interval vs goodput.

As shown in Figs. 28 and 29, E-SVM has lower dropping ratios and higher goodput than SVM for any time slot. For occurrence, at 0.2 s, E-SVM obtains a dropping ratio of 9.51698% and goodput of 46,775.9 b/s while SVM has a dropping ratio of 15.1124% and goodput of 13,768.8 b/s, indicating the better dropping and goodput of E-SVM.

**Fig. 30:**
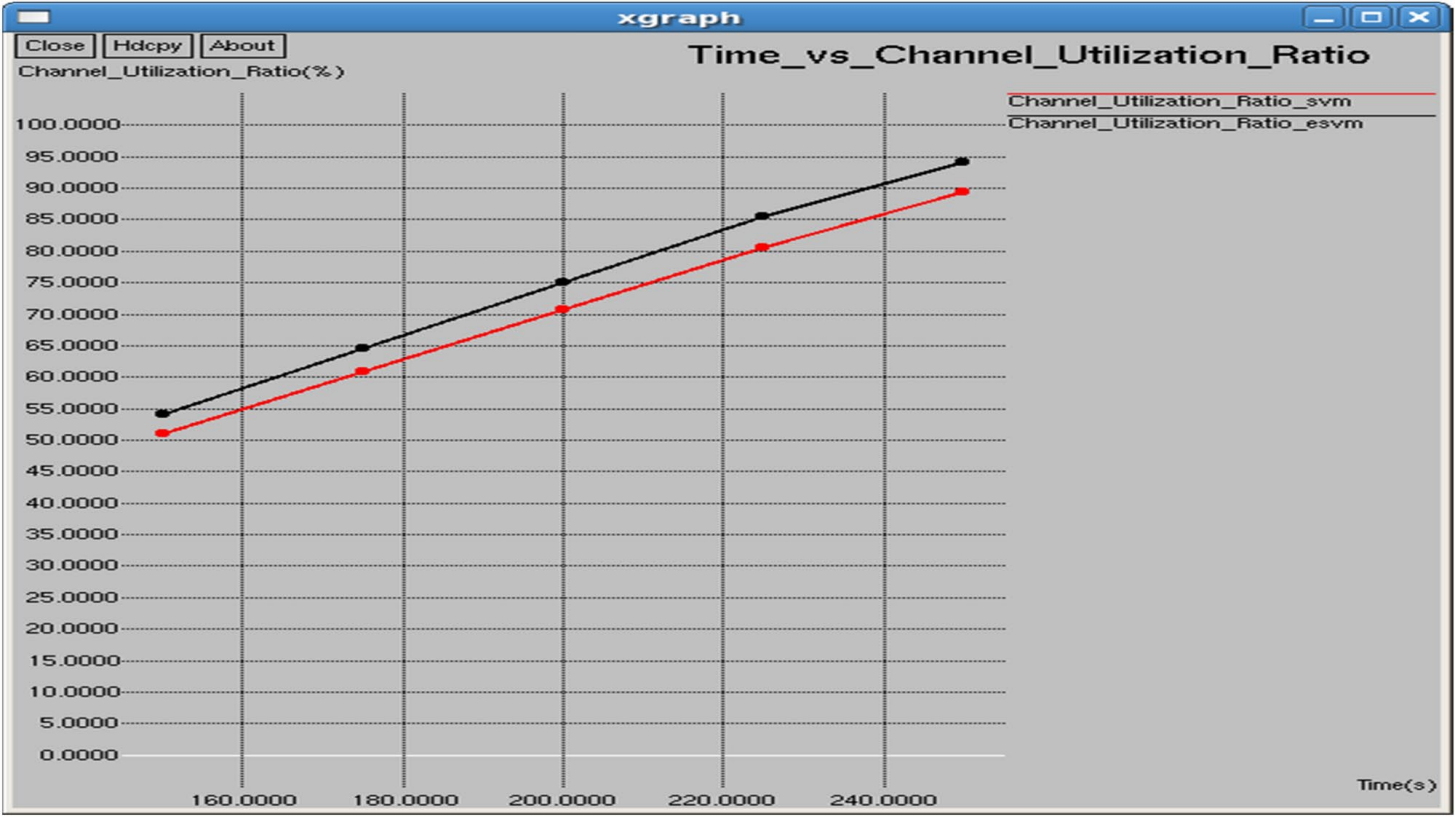
Time vs channel utilization ratio.

Table 3 and Fig. 30 illustrate the performance comparison between the proposed E-SVM and traditional SVM algorithms, specifically focusing on the channel utilization ratio. The graph illustrates that the channel utilization ratio for E-SVM is significantly higher compared to SVM across various periods.

**Table 3:** Performance Comparison of SVM and E-SVM for different times.

	Throughput (b/s)		Packets received (Packets)		PDR (%)		NRO (%)		Jitter (sec)		Goodput (b/s)	
TIME (s)	SVM	E-SVM	SVM	E-SVM	SVM	E-SVM	SVM	E-SVM	SVM	E-SVM	SVM	E-SVM
150	130,586	138,651	1101	1169	81.5556	86.5926	7.00636	6.57228	0.120628	0.114521	10,615.4	31,439.5
175	131,582	140,088	1315	1400	82.1875	87.5	6.82205	6.38714	0.120084	0.113482	11,107.3	33,289
200	132,309	140,790	1529	1627	82.6486	87.9459	6.69588	6.26245	0.119627	0.113014	11,453.4	34,731.8
225	133,092	141,248	1746	1853	83.1429	88.2381	6.58018	6.17539	0.119078	0.112737	11,775.4	35,792
250	134,470	142,122	1933	2043	84.007	88.7875	6.59493	6.20803	0.118247	0.112224	12,297.4	36,466.4

**Fig. 31:**
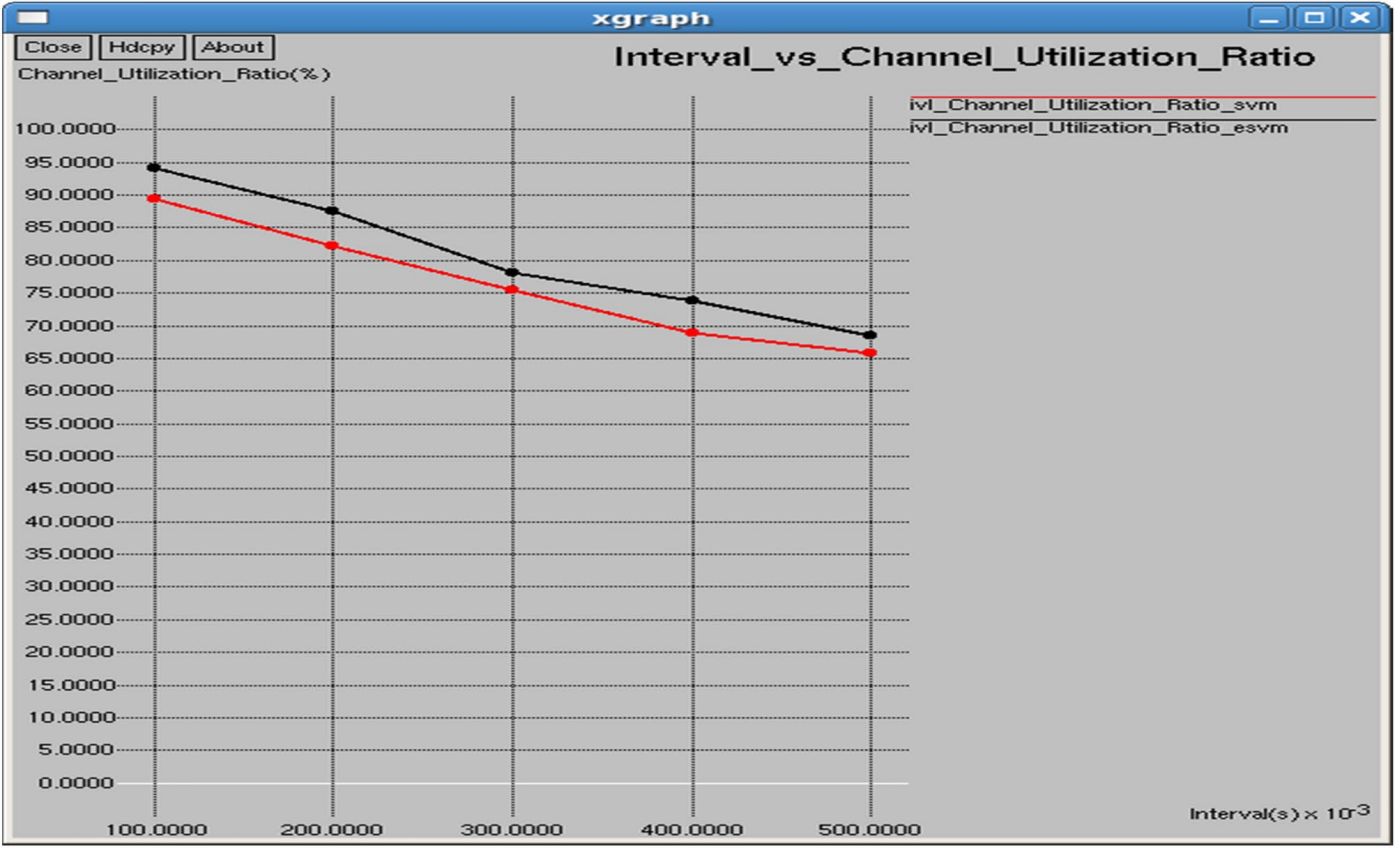
Interval vs channel utilization ratio.

Table 4 and Fig. 31 show the performance of the proposed E-SVM against SVM for Channel utilization ratio regarding interval. The graph reveals that the channel utilization ratio is consistently higher for E-SVM compared to SVM.

**Table 4:** Performance parameters for SVM and E-SVM with different times.

TIME (s)	Dropping ratio (%)		Delay (sec)		Control overhead (bits)		Average remaining energy (Joules)		Average consumed energy (Joules)	
	SVM	E-SVM	SVM	E-SVM	SVM	E-SVM	SVM	E-SVM	SVM	E-SVM
0.1	18.4444	13.4074	1.50725	0.508914	7714	7683	98.2918	98.9055	1.70815	1.09445
0.2	17.8125	12.5	1.44049	0.48064	8971	8942	98.0264	98.7409	1.97356	1.2591
0.3	17.3514	12.0541	1.39697	0.460673	10,238	10,189	97.758	98.5783	2.24201	1.42167
0.4	16.8571	11.7619	1.35877	0.447027	11,489	11,443	97.4979	98.4191	2.5021	1.58087
0.5	15.993	11.2125	1.30109	0.438759	12,748	12,683	97.363	98.2785	2.63699	1.72149

Table 4 demonstrates that E-SVM consistently outperforms SVM across multiple performance metrics, including dropping ratio, delay, control overhead, average remaining energy, and average consumed energy, at various time intervals. For instance, at the 0.1-s interval, E-SVM achieves a lower dropping ratio of 13.4074%, a reduced delay of 0.508914 s, a lower control overhead of 7683 bits, a higher average remaining energy of 98.9055 J, and a lower average consumed energy of 1.09445 J compared to SVM. This consistent superiority highlights the effectiveness of the proposed E-SVM approach in enhancing network performance.

**Fig. 32:**
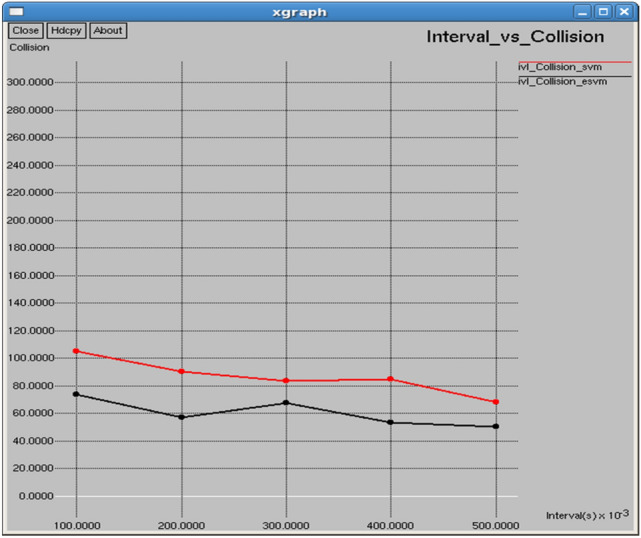
Interval vs collision.

**Fig. 33:**
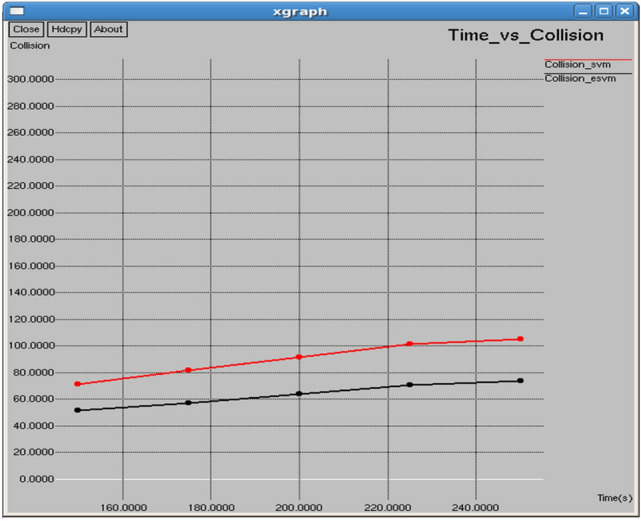
Time vs collision.

Table 5 and Fig. 32 illustrate the performance comparison between the proposed E-SVM and SVM regarding collision. The graph indicates that the collision rate is lower for E-SVM compared to SVM. Figure 33 demonstrates the performance of the proposed E-SVM against SVM for channel utilization ratio over time. The graph shows that the collision rate is also lower for E-SVM compared to SVM.

**Table 5:** Performance parameters for SVM, E-SVM with different intervals.

Interval (s)	Throughput (b/s)		Packets received (packets)		PDR (%)		NRO (%)		Jitter (sec)		Goodput (b/s)	
	SVM	E-SVM	SVM	E-SVM	SVM	E-SVM	SVM	E-SVM	SVM	E-SVM	SVM	E-SVM
0.1	134,470	142,122	1933	2043	84.007	88.7875	6.59493	6.20803	0.118247	0.112224	12,297.4	36,466.4
0.2	123,532	131,675	1775	1892	84.8876	90.483	7.12056	6.66966	0.129012	0.121145	13,768.8	46,775.9
0.3	113,013	116,910	1624	1680	84.7157	87.6369	7.74631	7.54226	0.140941	0.136611	14,342.4	38,580.4
0.4	102,483	110,136	1473	1583	83.2203	89.435	8.56076	8.02716	0.155389	0.144809	13,197.3	30,838.1
0.5	97,720.5	102,105	1404	1467	85.4534	89.2879	8.98006	8.60327	0.162768	0.156187	13,011.6	44,023.1

In every metric evaluated across all intervals presented in Table 5, E-SVM is always superior to SVM. As an example, at 0.1 intervals E-SVM provides more throughput (142,122 b/s) and gets more packets (2,043 packets), and has a better packet delivery ratio (88.7875%) and normalized routing overhead (6.20803%) and jitter (0.112224 s). The E-SVM gives a minimum goodput of 36,466.4 b/s compared to SVM. Across all time intervals, this trend continues with E-SVM demonstrating consistently better performance on networks than SVM.

Table 6 shows the comparison of SVM and E-SVM at different times. It can be observed that the E-SVM has a lower dropping ratio, low delay, less control overhead, high average remaining energy, and less Average Consumed Energy. Since the average remaining energy is high and the average consumed energy is low the lifetime of the network is increased.

**Table 6:** Performance parameters for SVM, E-SVM with different time intervals.

Interval (s)	Dropping Ratio (%)		Delay (sec)		Control Overhead (bits)		Average Remaining Energy (Joules)		Jitter (sec)	
	SVM	E-SVM	SVM	E-SVM	SVM	E-SVM	SVM	E-SVM	SVM	E-SVM
0.1	15.993	11.2125	1.30109	0.438759	12,748	12,683	97.363	98.2785	2.63699	1.72149
0.2	15.1124	9.51698	1.16205	0.342056	12,639	12,619	97.8602	98.5282	2.13982	1.47183
0.3	15.2843	12.3631	1.11558	0.414718	12,680	12,671	98.1279	98.4599	1.87212	1.54014
0.4	16.7797	10.565	1.21237	0.518839	12,810	12,707	98.1677	98.5234	1.83229	1.47657
0.5	14.5466	10.7121	1.22967	0.363446	12,658	12,621	98.263	98.7954	1.73702	1.20463

## Conclusion

This work aims to propose a model to address blackhole and wormhole attacks in wireless ad-hoc networks. The cross-layer capabilities are provided through three layers: the network layer, the MAC layer, and the physical layer, independent of network protocols. The network’s lifetime is improved, as discussed in the results and discussion section. The performance of the proposed model has been evaluated in terms of average consumed energy, average remaining energy, packets received, packet delivery ratio, delay, jitter, throughput, normalized overhead, dropping ratio, and goodput over time intervals. The proposed model excels in energy-efficient, reliable data communication, achieving high throughput (116,910 b/s) and PDR (89.2879%) with minimal delay (0.508 s) and packet loss (9.517%), ideal for real-time applications. Its efficient bandwidth use (46,775.9 b/s goodput) and high energy sustainability (98.9055 J remaining) ensure prolonged network viability in constrained environments. The channel utilization ratio demonstrates that isolating nodes during an attack minimizes the level of the attack. The proposed model achieved outstanding results with E-SVM which is comparatively better than existing methods.

The future work will build upon this framework by integrating it into Fifth Generation (5G) networks, demonstrating its relevance and application to future communication generations. This integration aims to leverage 5G low-latency, high-speed, and massive connectivity, thus enhancing model efficiency in diverse environments. Testing under more complex attack scenarios, such as Artificial Intelligence (AI)-based attacks, combined attacks at different nodes, or exploits of weaknesses in 5G, will demonstrate the robust nature of the framework and its adaptability. Investigating these methods will ensure that this model aligns with the latest network schema while deepening our understanding of stimulating the next-generation wireless communication system against emerging threats.

## Data Availability

The authors confirm that the data supporting the findings of this study are available within the article.
